# Supplementary material for: Filling the gaps between tide gauges: Demonstrating high-resolution seasonal high tide flooding predictions using NOAA’s Coastal Ocean Reanalysis
Source: PLoS One. 2026 Mar 30;21(3):e0344695. doi: 10.1371/journal.pone.0344695 (PMC13035166; doi:10.1371/journal.pone.0344695)
Supplement: S1 Appendix — (DOCX) [file pone.0344695.s001.docx]

**S1 Appendix**

The accuracy of CORA-derived HTF predictions was very similar at three month lead as at one month lead. For example, values of HL agreement, bias, and MAE at three month lead were nearly all identical to those at one month lead (compare Table S1 to Table 1). Using the CRPS, performance of the HTF model declined when CORA input was used instead of gauge data by the same amount at three month lead as at one month lead (5%). Further, on average across the considered flood thresholds, CORA-derived HTF predictions were skillful at nearly the same proportion of stations for which gauge-derived predictions were also skillful at three month lead (96%) as at one month lead (94%). Additionally, performance of the HTF model in terms of BSS declined by the same amount when CORA was used instead of gauge data at three month lead as at one month lead (2%). Finally, reduction in AUC for CORA input was nearly the same at three month lead (2%) as at one month lead (1%).

Table S1. Average values of HL agreement, bias, and MAE, respectively, within each region and for each flood threshold at three month lead.

| Region | $MHHW+0.15$ m | $MHHW+0.30$ m | $MHHW+0.45$ m | $MHHW+0.60$ m | **mean** |
| --- | --- | --- | --- | --- | --- |
| NE | 76% \| 0.22 \| 0.29 | 87% \| 0.11 \| 0.16 | 94% \| 0.01 \| 0.07 | 97% \| -0.01 \| 0.03 | **89% \| 0.08 \| 0.14** |
| MA | 79% \| 0.14 \| 0.23 | 82% \| 0.08 \| 0.19 | 98% \| 0.01 \| 0.03 | 100% \| 0.00 \| 0.00 | **90% \| 0.06 \| 0.11** |
| SE | 81% \| 0.07 \| 0.20 | 90% \| 0.04 \| 0.11 | 97% \| 0.00 \| 0.03 | 100% \| 0.00 \| 0.00 | **92% \| 0.03 \| 0.08** |
| EG | 77% \| 0.02 \| 0.25 | 85% \| 0.01 \| 0.16 | 98% \| 0.01 \| 0.02 | 100% \| 0.00 \| 0.00 | **90% \| 0.01 \| 0.11** |
| WG | 68% \| -0.10 \| 0.36 | 63% \| -0.29 \| 0.49 | 85% \| -0.12 \| 0.18 | 99% \| -0.01 \| 0.01 | **79% \| -0.13 \| 0.26** |
| **mean** | **76% \| 0.07 \| 0.27** | **81% \| -0.01 \| 0.22** | **94% \| -0.02 \| 0.07** | **99% \| 0.00 \| 0.01** |  |
