# Supplementary material for: Filling the gaps between tide gauges: Demonstrating high-resolution seasonal high tide flooding predictions using NOAA’s Coastal Ocean Reanalysis
Source: PLoS One. 2026 Mar 30;21(3):e0344695. doi: 10.1371/journal.pone.0344695 (PMC13035166; doi:10.1371/journal.pone.0344695)
Supplement: S4. Appendix — (DOCX) [file pone.0344695.s004.docx]

**[S4 Appendix.](https://docs.google.com/spreadsheets/d/1Lcg_6__o4PXZAOukKMs-Yd6rwOJj13PmDI2kUBXZkEw/edit?usp=sharing)**

Agreement between CORA-derived and gauge-derived HTF predictions was found to be particularly weak at Grand Isle, L.A. and Rockport, T.X. Further analysis revealed systemic errors in CORA water levels and derived tide predictions at these locations, as will be described further in this appendix and shown in Figures S4-1 and S4-2.


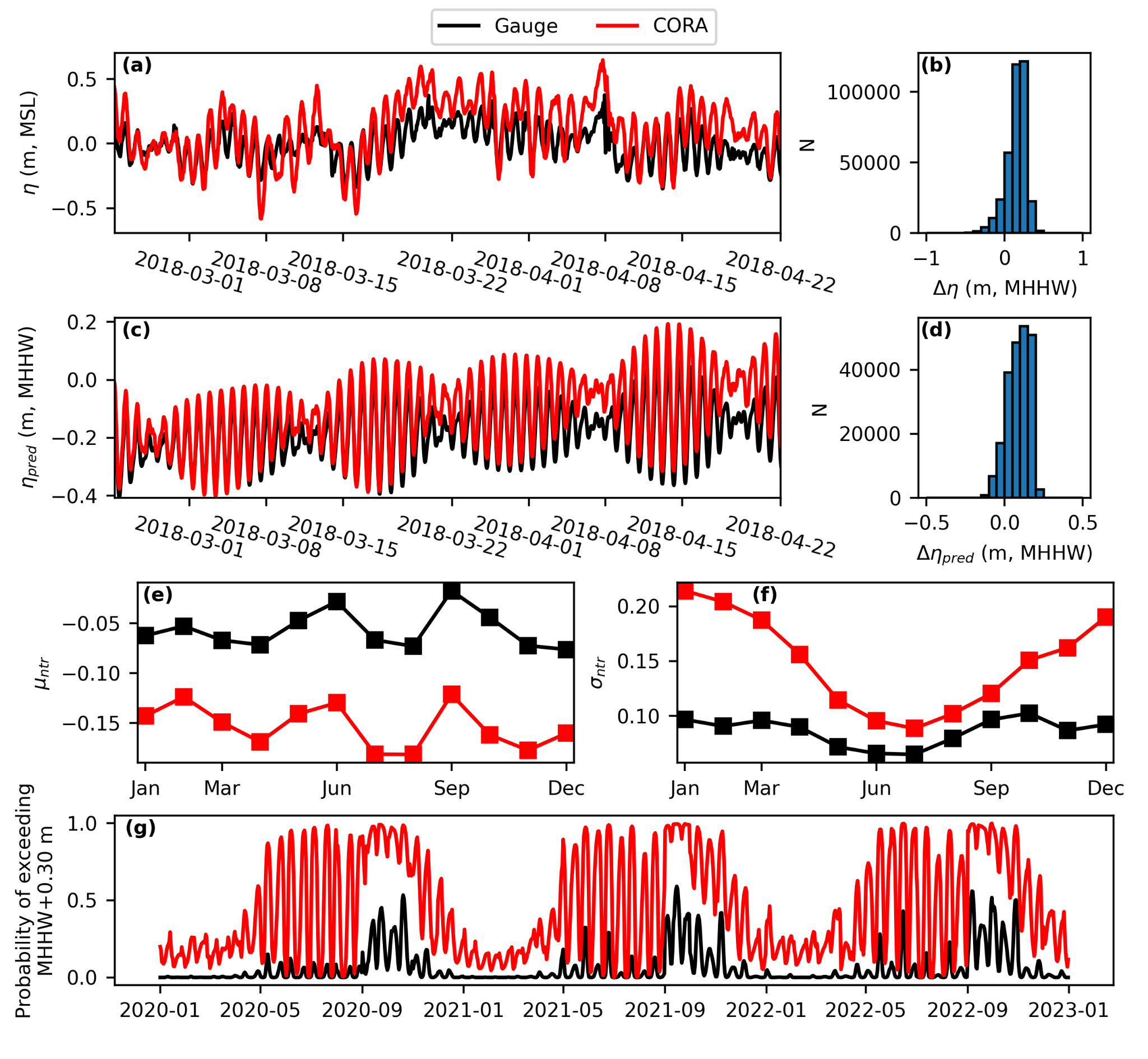


Figure S4-1. Explanation of differences between CORA-derived and gauge-derived HTF model performance at Grand Isle, L.A. (a) Observed hourly water levels during an example period. (b) Distribution of hourly errors in CORA water levels for the full training period, 1997 through 2021. (c) Hourly tide predictions during an example period. (d) Distribution of hourly errors in CORA tide predictions for the full training period, 1997 through 2022. (e) Monthly mean non-tidal residual. (f) Standard deviation around the monthly mean non-tidal residual. (g) The resulting daily probability of high tide flooding above $MHHW+0.30$ m.

At Grand Isle, CORA water levels exhibit a larger range than observed water levels. At times, CORA low waters are lower than observations, other times higher, while high waters are frequently overestimated (Figure S4-1a). When transferred to the derived MHHW datum, which is within 2 cm of the observed value, CORA water levels are biased high by 13 cm on average and the distribution of differences is left skewed (Figure S4-1b). The differences in water levels result in CORA-derived tide predictions with over-estimated tide range and a high bias, by 9 cm on average though particularly for high tides, and a left-skewed distribution of differences relative to gauge-derived tide predictions (Figure S4-1c,d). The combination of these errors leads to low-biased $\mu_{NTR}$ for CORA by ~10 cm (Figure S4-1e) and CORA-derived $\sigma_{NTR}$ which is biased high relative to the gauge by as much as 100% during January and December (Figure S4-1f). While the high-biased tide predictions and low-biased $\mu_{NTR}$ largely offset each other in the HTF probability computation, the larger $\sigma_{NTR}$ increases CORA-derived HTF probability at all times relative to the gauge (Figure S4-1g).

At Rockport, a systemic high bias of 11 cm on average is observed in CORA water levels on MSL (Figure S4-2a). Yet, the computed MHHW value for CORA is <1 cm different from the published value at the gauge. Therefore, the high bias remains when CORA water levels are transferred to MHHW (Figure S4-2b) and propagates to the derived tide predictions, which are biased high by 17 cm on average while tidal range is underestimated (Figure S4-2c,d). While $\sigma_{NTR}$ are similar in this case (Figure S4-2f), these differences lead to CORA-derived $\mu_{NTR}$ that is biased low by 7 cm (Figure S4-2e). The high bias in the tide predictions, which is larger than the low bias in $\mu_{NTR}$, increases CORA-derived HTF probability at all times relative to the gauge (Figure S4-2g). Note that this is a similar end-result to Grand Isle though arises through a different mechanism.


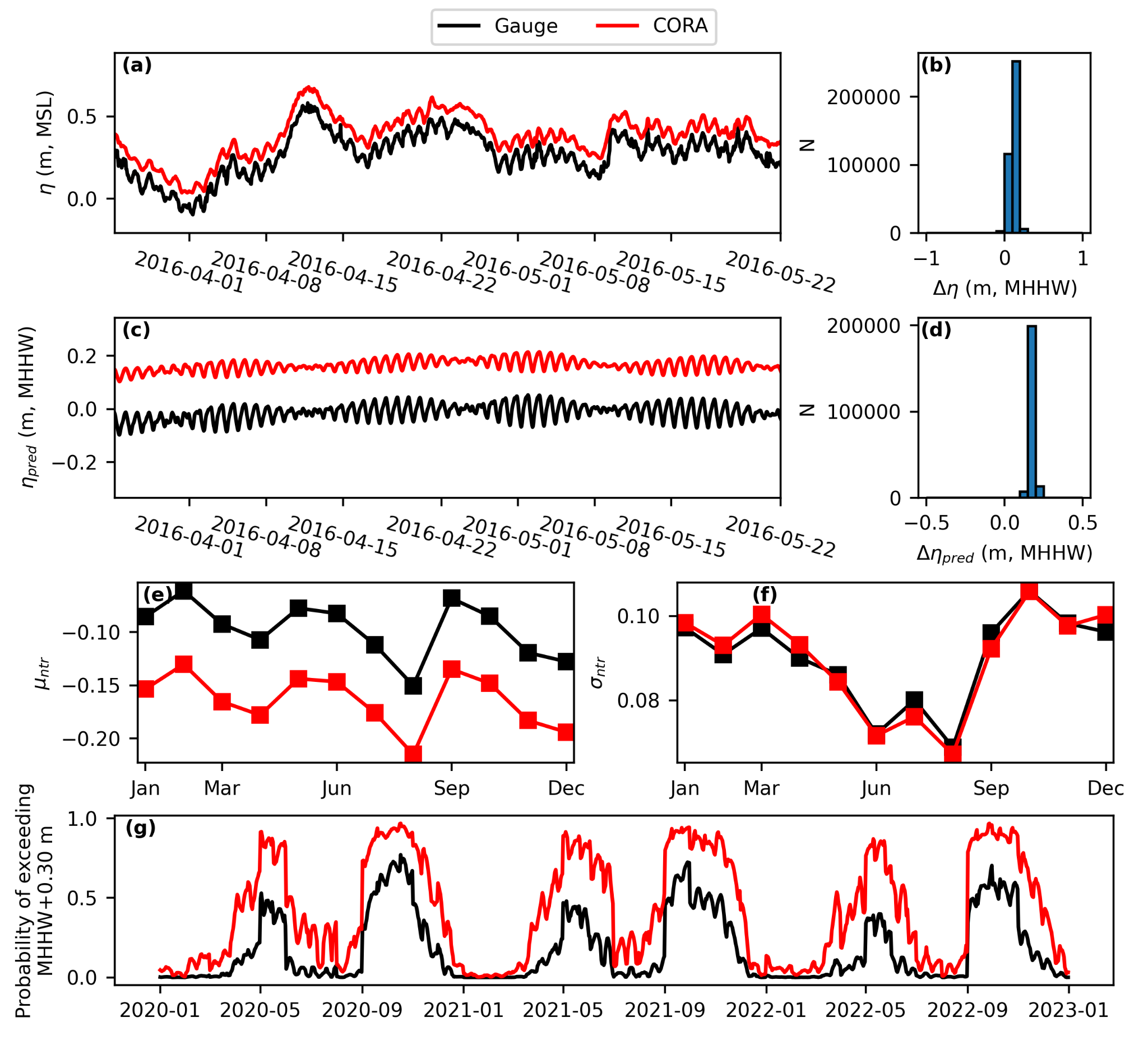


Figure S4-2. Explanation of differences between CORA-derived and gauge-derived HTF model performance at Rockport, T.X. (a) Observed hourly water levels during an example period. (b) Distribution of hourly errors in CORA water levels for the full training period, 1997 through 2021. (c) Hourly tide predictions during an example period. (d) Distribution of hourly errors in CORA tide predictions for the full training period, 1997 through 2022. (e) Monthly mean non-tidal residual. (f) Standard deviation around the monthly mean non-tidal residual. (g) The resulting daily probability of high tide flooding above $MHHW+0.30$ m.
