# Supplementary material for: Filling the gaps between tide gauges: Demonstrating high-resolution seasonal high tide flooding predictions using NOAA’s Coastal Ocean Reanalysis
Source: PLoS One. 2026 Mar 30;21(3):e0344695. doi: 10.1371/journal.pone.0344695 (PMC13035166; doi:10.1371/journal.pone.0344695)
Supplement: Attachment — Submitted filename: ConlinEtAl_PLOSOne_V2_R2R.pdf [file pone.0344695.s005.pdf]

We thank the Editor and two anonymous Reviewers for their comments. Below, please find the original comments in **bold** and our responses in plain text**.** Line numbers correspond to the new clean version of the manuscript.

<u>Additional Editor Comments:</u>

The paper needs a minor revision.

The minor revision has been completed following the Reviewers' suggestions, as detailed below.

<u>Reviewers' comments:</u>

Reviewer's Responses to Questions

1. Is the manuscript technically sound, and do the data support the conclusions?

The manuscript must describe a technically sound piece of scientific research with data that supports the conclusions. Experiments must have been conducted rigorously, with appropriate controls, replication, and sample sizes. The conclusions must be drawn appropriately based on the data presented.

Reviewer #1: Yes

Reviewer #2: Yes

No revisions were required for this comment.

---

2. Has the statistical analysis been performed appropriately and rigorously?

Reviewer #1: Yes

Reviewer #2: Yes

No revisions were required for this comment.

---

3. Have the authors made all data underlying the findings in their manuscript fully available?

The [PLOS Data policy] requires authors to make all data underlying the findings

described in their manuscript fully available without restriction, with rare exception (please refer to the Data Availability Statement in the manuscript PDF file). The data should be provided as part of the manuscript or its supporting information, or deposited to a public repository. For example, in addition to summary statistics, the data points behind means, medians and variance measures should be available. If there are restrictions on publicly sharing data—e.g. participant privacy or use of data from a third party—those must be specified.

Reviewer #1: Yes

Reviewer #2: Yes

No revisions were required for this comment.

---

4. Is the manuscript presented in an intelligible fashion and written in standard English?

PLOS ONE does not copyedit accepted manuscripts, so the language in submitted articles must be clear, correct, and unambiguous. Any typographical or grammatical errors should be corrected at revision, so please note any specific errors here.

Reviewer #1: Yes

Reviewer #2: Yes

No revisions were required for this comment.

---

5. Review Comments to the Author

Reviewer #1: The article is well written and tackles the important topic of predicting risks of coastal flooding for months ahead. The frequency of these events is rapidly increasing along all coastal locations, and particularly for locations with high rates of relative sea level rise. Several of the authors had worked on and published a related paper presenting a new method to predict monthly high tide flooding. The previous work was somewhat limited spatially to tide gauge locations. The present work extends substantially the spatial span of the predictive model by using the relatively recent oceanographic CORA reanalysis data set.
The new method extends the previous work of estimating daily cumulative flood probabilities for tide gauges based by using the CORA data. The article makes a well documented argument that the CORA reanalysis data set can be used to predict monthly high tide flooding, at least for most locations.
I have two reservations regarding the paper. The authors are very knowledgeable and already discuss these limitations on a few occasions but, if the authors agree, I think these limitations should be pointed out more explicitly including in the discussion and conclusion. These limitations were already present when the method only focused on

tide gauge data but the new method based on CORA reanalysis increases greatly the spatial extent of the predictions and now includes more locations with complex bathymetry within shallow bays, lagunas, and estuaries. Figure 7 provides a good example of how the CORA based method expands substantially the area for the predictions.

We thank the Reviewer for their positive outlook on this paper as well as their astute and helpful comments. Below, we detail how we have addressed each of the Reviewer's specific comments. As shown below, we have now more explicitly pointed out limitations of the work in the Discussion and Conclusions.

(1) The spatial resolution of the CORA data set is 400-500m. This resolution is not a concern for the open coast and large deep bays but I think this resolution is too coarse to accurately model some locations in bays and estuaries, particularly where water level dynamic is influenced by shallow passes with widths in the range of a few meters to even 10-20m. If this is indeed a limitation, the article should include a more specific discussion that the method should be used carefully and possibly not at all for shallow coastal areas including those influenced by shallow passes. Some related precautions were taken for the modeling example near Charleston using points 500m away from the shoreline and I agree with the related great benefits discussed on lines 490-493. If the authors agree I think they need to be more explicit maybe stating in the conclusion that the method should be used only for coastal areas where modeling at a 400-500 m resolution does not significantly influence water level dynamic. It could be added that a reanalysis at a higher resolution would narrow this limitation.

The Reviewer is absolutely correct about the improved though still limited resolution of CORA and its potential impact on HTF predictions. In response to this comment, as well as similar comments from Reviewer #2, we have added a new paragraph to the section entitled "Towards an operational community-level HTF outlook" (L587-612) more explicitly and deeply discussing the limitations of the NWLON-based validation used in this work.The Reviewer's points in this comment have been integrated into that paragraph, for example where we now state:

> "The 400-500 m resolution of CORA is also an important consideration: it is possible that narrow waterways such as back-barrier bays and inland estuaries with hydrodynamics varying on spatial scales of O(1-10 m) may not be captured sufficiently or at all by CORA, leaving HTF predictions not possible in these locations." (L600-603)

We have also added the following sentence to the Conclusions to further summarize this point:

> "While further research is needed to more comprehensively understand the accuracy of CORA-derived predictions in all relevant locations and morphodynamic environments…" (L663-665)

Further, as suggested, we have now noted that improved spatial resolution could help alleviate this limitation:

> “Improving the spatial resolution in future CORA versions could help alleviate this limitation.” (L603-604)

More relevant details are also provided in our response to the Reviewer’s comment two below this one.

(2) The method does not work as well in the Gulf, Eastern (EG) and Western Gulf (WG). This is not a big concern given the benefits, but I would like to have more details on the locations for which the assessment was made and a more extensive and more precise discussion as to areas of the Gulf where the method should not be considered as accurate and possibly not be applied. The limitations are clearly shared and discussed for the tide gauges of Grande Isle, Louisiana and Rockport, Texas in Appendix S4. I would like to see more explicit results for all the locations of the EG and WG similar to the Table in appendix S1. In particular, I am wondering how many locations were used to compute the EG and WG averages. For the WG, the tide gauges of Corpus Christi and Sabine Pass were removed for data quality concerns (line 212) as well as Rockport (line 381) "where errors in CORA water levels and tide predictions led to strong overprediction of HTF probabilities". I would like to know more explicitly which stations were left for the assessment, clearer information than presently in the text and in Appendix S2.

Though we feel that Figures 1 and 4-6 already quite clearly show the stations in the EG and WG that were included in the analysis by showing (Figure 1) and listing (Figures 4-6) all of the individual stations in all regions, we have made two changes to address this comment. First, we have added a column to Table 1 called “N” which explicitly lists the number of stations used to compute the statistics in each region (including assimilated vs. unassimilated), directly addressing the Reviewer’s particular question. Second, we have added a new supplement (now S3 Table) that lists the specific quantitative results for each station included in the analysis, including those stations in the Gulf. The precise number of stations used to compute the averages within each region and/or their names are now apparent in numerous locations: Figure 1, Table 1, Figures 4-6, and S3 Table.

We also want to clarify that Corpus Christi and Sabine pass were not removed for data quality concerns, as the Reviewer states, but because of “incomplete data records” (L212-213). The quoted section in the Reviewer’s comment applies only to Grand Isle and Rockport as stated on L392-394.

Other less important to me detail type question regarding lines 102-110. The method was setup for tide gauges, i.e. for locations that were selected to typically not go dry and benefit from some water depth during most of the year. The CORA data set allows to expand the predictions to coastal areas including some which will, at times, have very little water depth. For shallow areas, the tidal dynamic and potential nonlinear interactions between the non tidal component and the tidal dynamic could affect the distributions, e.g. the timing of the tides will change based on non tidal water level component. Also could this affect the estimation of the dampening for locations with at times very low water levels? As the work focusses on the higher deciles of water levels

this is likely not significant but wondering how relevant it is and if it should be further discussed. Note that the largest differences between CORA and tide gauged based predictions are for low water level predictions and I am concerned about areas that have, at times, water levels lower than that of nearby tide gauges and may not be as accurately modeled using CORA.

The Reviewer raises an important point here, which is related to their point (1) about CORA's accuracy in shallow and narrow waterways. Within the new limitations paragraph (L587-612), we now acknowledge and discuss this comment's core idea relating to non-linear and non-Gaussian processes in shallow areas by noting:

> "Even if sampled, however, such environments may not be inundated at all times and are likely very shallow, which could drive nonlinear interactions between water level components that result in strongly non-Gaussian distributions [52]." (L604-607)

And we also now acknowledge the need for further study on this topic by adding:

> "Since the HTF model was developed for always-inundated tide gauge data, we here limited the application to CORA nodes that were (nearly) never dry. Further research is needed to develop methods to handle intermittently dry data and further explore possible non-Gaussian distributions in these shallow CORA locations." (L607-610)

Indeed, recent work that we have now cited in the paper (L612) highlights that non Gaussian distributions may better characterize non-tidal residuals even at tide gauges, supporting the Reviewer's comment and the importance of our additions to the text.

In line 284-285, the authors share that "The RMSE of CORA-derived trends relative to the published values at the NWLON stations was 1.27 mm/yr, however not considering Eagle Point, TX the RMSE was 0.74 mm/yr (S3 Appendix)." Eagle Point should not be used for rslr trends during that period as it was strongly impacted by oil and gas extraction leading to large changes, increasing and decreasing, in land subsidence and a definitely non linear rslr trend (there is a paper documenting rslr changes and their driver for that location, I can find it if helpful).

We thank the Reviewer for pointing out this applicable previous research at Eagle Point, which we were able to locate. We now acknowledge this previous work in the Results as the reason that CORA's trend is quite different from observations at this location, noting:

> "Eagle Point, TX has been strongly impacted by land subsidence due to oil and gas extraction [46], processes CORA does not capture at this unassimilated station (Fig. 2c)." (L362-363)

We have also now added this to the discussion of why CORA-derived HTF performance is relatively weak at some Gulf locations, noting:

> "Additionally, there are Gulf locations with relatively large local rates of land subsidence (such as Eagle Point, T.X.; [46]), which CORA cannot capture at unassimilated locations.

> Grand Isle and Rockport in particular (S4 Appendix), as well as the difference in trend at Eagle Point (Fig. 2c), highlight these inaccuracies in CORA." (L540-543)

Line 587: Great to mention future modeling work to provide calibrated uncertainty bounds on HTF predictions. Big endeavor but would be most helpful to the users.

We thank the Reviewer for their positive outlook on our proposed probabilistic approach. Note that we have added additional details about the approach in response to comments by Reviewer #2.

<u>Reviewer #2</u>: This manuscript assesses the ability of a water level reanalysis, CORA, to reproduce high tide flooding (HTF) predictions generated from tide gauge (TG) data (using the methodology of Dusek et al 2022). A pilot example of the degree of spatial variability of high tide flooding metrics around Charleston, South Carolina is presented. Finally, the authors give an example of how uncertainty in CORA might be introduced into the HTF predictions.
This is a "methods" paper, and is a useful analysis that may help serve to extend the spatial coverage of HTF predictions. The analysis is original, well-described, and clearly executed -- so is technically sufficient for publication. I have 4 substantive comments that would improve the paper. I am not wed to a particular approach to achieve these goals, but I think they should be carefully considered by the authors.

We thank the Reviewer for their accurate summary of the paper and their helpful comments. As detailed below, we have addressed each of the Reviewer's four substantive comments as well as their minor comments.

Substantive issue 1. The danger in this analysis is that CORA does well at tide gauge locations (which are constrained by data assimilation), but poorly elsewhere, where data assimilation is a weaker constraint. The authors do a good job showing that there are not dramatic differences in CORA v. TG HTF predictions at assimilating and non-assimilating tide gauges, which helps their argument. However, there are more assimilating tide gauges than non-assimilating, and substantial differences in the spatial distribution of non-assimilated tide gauges evident in Fig. 1. For example there seem to be a lot of non-assimilated tide gauges in the Chesapeake Bay, and very few over the entire SAB. In addition, the tide gauges that aren't assimilated appear to be upstream and in problematic locations. In some sense this might be a *good* thing, because CORA seems to work even in these problematic sites. EXCEPT for Grand Isle and Rockport, which are thrown out of the analysis. A physical understanding of these differences across non-assimilating TGs would help inform where the use of CORA might be problematic. I have the following suggestions that the authors might pursue to bring these concerns to light, and, if possible, alleviate them:

The Reviewer raises an important point about limitations to the generalizability of our results- even for non-assimilated stations- to all possible locations and morphodynamic environments. Reviewer #1 raised similar concerns though more specifically about very shallow and dynamic environments. We have added a paragraph to the section entitled "Towards an operational community-level HTF outlook" (L587-612) more explicitly and deeply discussing the limitations of the NWLON-based validation used in this work. We feel that this new paragraph provides the insight needed to better understand where CORA-derived predictions may be problematic and where we are still not able to know for sure without further research. Therein we have followed the following four suggestions by the Reviewer, as detailed further below.

> **a. Provide more details about the non-assimilated TG comparison. Explain the rationale for which tide gauges are assimilated and why, and whether we think the non-assimilated TG's are representative. Perhaps include another figure which shows**

more detailed results at non-assimilating sites (also see my thoughts on substantive issue 2).

We have added a more detailed description of the gauges that are and are not assimilated. Indeed, the Reviewer is correct that non-assimilated tide gauges are in non-open coast environments. This is now stated explicitly:

> "Importantly, only open-coast stations are used in the data assimilation, meaning that validation (unassimilated) stations are in non-open coast (riverine, estuarine, and bay) environments." (L151-153)

Further, as also suggested in substantive issue 2, we have now made what was Appendix S3 a figure in the main text (now Figure 2) and have differentiated between assimilated and unassimilated stations in that figure. A new results section (L354-363) has been added to report the results in the figure.

Finally, we have added a column in Table 1 (also in response to a comment by Reviewer #1) which explicitly states the number of assimilated and unassimilated stations in each region included in the analysis. We have also added S3 Table which shows the values for each station individually, including its assimilation status.

b. Carefully choose words about what is demonstrated by the non-assimilated gauges (I think for the most part the authors did a good job).

We have added further clarifications in the text about what the comparison at non-assimilated gauges does and does not demonstrate. For example, in the paragraph discussing the unassimilated results we have added:

> "In particular, since unassimilated stations are located in non-open coast environments [24] (see also; Fig. 1), accuracy at unassimilated stations may be somewhat representative of accuracy specifically in these environments." (L517-520)

We have also added the following in the new limitations paragraph:

> "For example, even though non-assimilated stations are in non-open coast environments, they do not sample back-barrier locations or locations very far up rivers, leaving the accuracy of CORA-derived HTF predictions in these environments technically unknown." (L589-592)

c. There should be some mention in the discussion about the potential limitations of CORA, and potential next steps that could truly support confidence in predictions at non-TG locations. A true out-of-sample validation exercise would be a difficult task as it might need a non-data assimilating appropriate hydrodynamic model, or by some other on-the-ground metric of flooding. I don't think this is necessary in order to publish this paper but some discussion of limitations and possible alternative validation exercises would help. (I wonder if there is a free running version of CORA

that could be used to assess the effect of assimilation on water level variability away from TGs?).

In the new limitations paragraph, we have captured the suggestions in this particular comment about limitations and future validation work by adding:

> "For example, even though non-assimilated stations are in non-open coast environments, they do not sample back-barrier locations or locations very far up rivers, leaving the accuracy of CORA-derived HTF predictions in these environments technically unknown. While potential validation data in these and other environments is inherently limited by a lack of observations (hence the need for CORA), non-NWLON tide gauge networks such as those recently available through Hohonu Inc. [51] as well as supporting data such as media reports of flooding occurrences could be used to more fully assess CORA-derived HTF prediction accuracy at new locations in future work. Additionally, a more bottom-up approach could be an analysis of pre- and post-assimilated CORA output to deduce a more robust understanding of the spatially variable effects of the data assimilation as a function of morphodynamic environment and/or distance from NWLON stations." (L589-600)

We have also included a note in the Conclusions regarding the need for further work to more fully validate CORA-derived HTF predictions at new locations:

> "While further research is needed to more comprehensively understand the accuracy of CORA-derived predictions in all relevant locations and morphodynamic environments…" (L663-665)

d. The appendix discussing Grand Isle and Rockport analyses how the statistics differ but does not really address why. A more complete description of the processes underlying differences, including their spatial scale, would help the reader (or future implementers) decide what generic types of locations might be problematic.

We agree with the Reviewer that providing an explanation for CORA's limitations at Grand Isle and Rockport is important. While S4 Appendix does not address this, as the Reviewer notes, it was already addressed - at least to some extent- in the Discussion, where we noted that "It is noteworthy that there are known limitations to CORA in the Gulf: available data from NWLON stations to drive the data assimilation are particularly scarce here (see Fig. 23 in [24]), causing water level errors relative to typical variability to be especially large (see Fig. 27 in [24])." (now L535-538). We have now added even more details regarding the reasons for CORA's limited performance in the Gulf in response to other comments by both Reviewers. For example, we now note how highly localized rates of VLM in the Gulf cannot be adequately captured in CORA:

> "Additionally, there are Gulf locations with relatively large local rates of land subsidence (such as Eagle Point, T.X.; [46]), which CORA cannot capture at unassimilated locations." (L540-542).

Regarding the Reviewer's request to include information on the spatial scale of processes driving differences, it is difficult from the analyses in this work to speak to this directly and specifically. While this analysis examines errors at specific tide gauge locations, the spatial propagation of these errors between tide gauges will be mediated by an array of factors such as bathymetry, morphodynamic environment, proximity of neighboring tide gauges, and the spatially variable impacts of the data assimilation (as the Reviewer noted previously) on model output. We do not have enough information to provide explicit guidance on these relationships in this work, as the Reviewer has themselves alluded to. However, our inclusion of the new limitations paragraph (L587-612) more explicitly and deeply discusses the limitations of the NWLON-based validation used in this work. We feel that this new paragraph provides the insight needed to better understand in general where CORA-derived predictions may be problematic and lays out where we are still not able to know for sure without further research. For example, specifically regarding the question of the spatial scale of errors and processes, the following sentence has been added:

> "Additionally, a more bottom-up approach could be an analysis of pre- and post-assimilated CORA output to deduce a more robust understanding of the spatially variable effects of the data assimilation as a function of morphodynamic environment and/or distance from NWLON stations." (L597-600)

We also now discuss spatial scales of accuracy in CORA in relation to its 400-500 m resolution in response to comments by Reviewer #1 (L600-604).

Substantive issue 2. My second comment concerns the lack of clarity what driver of sea level variability (linear SLR, tides, etc) is causing the discrepancy between CORA and TGs in different locations. I am very interested in seeing these deviations by tide gauge location (similar to what is done in Appendix 4). It looks like appendix S3 is a good start. At the very least, I would consider moving that figure into the main text. You could also add more detail, i.e. color coding points by region, and using square and circles (as in figure 1) to differentiate between assimilating and non-assimilating tide gauges. Note that even if the RMSEs/biases are calculated it's difficult to identify how these cascade into errors in flood probabilities.

We agree with the Reviewer that what was Appendix S3 is a concise way to examine the drivers of performance differences at different stations. As suggested, what was Appendix S3 has been moved to the main text (now Figure 2) and more detail has been added by differentiating between assimilated and unassimilated stations and coloring by region. A new Results section (L354-363) has been added to report the results in the figure.

Substantive issue 3. How does vertical land motion at tide gauges enter into CORA? Is it all driven by the data assimilation at tide gauges, and thus contains both an ocean steric/mass change and VLM? I think it is worth including some text into this paper on where those changes arise. They have very different implications for the spatial scales over which long term rates (or even nonlinear) changes can be extrapolated. It would also help interpret the deviations shown in Fig. S3.

The Reviewer is correct that VLM enters CORA through the data assimilation, meaning CORA contains both eustatic and local VLM signals. As suggested, this information has been added to the text where CORA is described. We now note:

> "CORA therefore represents a reanalysis of local relative sea levels driven by both eustatic sea level changes and spatially variable rates of vertical land motion." (L148-150)

Substantive issue 4. I would prefer to have the entire analysis/results communicated using the probabilistic approach rather than just an add-on at the end (in fact, a probabilistic propagation of uncertainty should probably be used for the TG-based HTF predictions as well). However, if the paper stays in the same format, the methodology for the probabilistic presentation of results (Figure 9) should be conveyed in the methods section, even if just sketched out. There needs to be at least a few equations describing which terms in the HTF model are assigned uncertainties, and how those are assigned.

We agree with the Reviewer that probabilistic HTF predictions are a worthy goal in the long-term. However, our goal with this paper was to compare CORA-derived HTF predictions with gauge-derived predictions. Using CORA within the published HTF modeling framework was deemed the most straightforward insightful way to do this, rather than adding additional complexity to the comparison through introduction of the probabilistic approach such as choice of ensemble percentile to use in the comparison. Further, the approach is relatively inefficient and may not be the optimal approach to use, and additional research is required to further develop the method. Therefore, we decided to present the method within the Discussion to illustrate it as just one possible approach to account for uncertainty and serve as the basis for future research on the topic, rather than muddying the core comparison between CORA-derived and tide gauge-derived HTF predictions in the main analysis. Since the core analysis of the paper does not utilize the probabilistic approach, we feel it is most appropriate to keep the presentation of the method in the Discussion section. However, as suggested by the Reviewer, we have added details (including an equation) regarding the terms that are assigned uncertainties and how they are assigned:

> "For example, by examining CORA vs. observed NTR, Eq. (1) can be modified as:
>
> $$\mu_{clim}(t) = \mu_{month}(t) + \mu_{tide}(t) + p(t) + \delta_{NTR}, \qquad (7)$$
>
> where δ_{NTR} is a random value between 0 and the standard deviation of the CORA NTR errors. The modified value of $\mu_{clim}(t)$ is then compared with the distance between the flood threshold and predicted tide similarly modified within the standard deviation of the CORA tide prediction errors δ_{tide}." (L632-639)

We have also added a sentence to clarify that this approach requires further research and refinement:

> “However, the iterative nature of the method is relatively inefficient, and other approaches should also be investigated to facilitate application at the many thousands of possible CORA nodes.” (L645-647)

Other minor comments are below:
30: the basis for -> an approach to generating

This suggestion has been implemented. (L30)

36: remove also

This suggestion has been implemented. (L36)

84: see line 30 comment

This suggestion has been implemented. (L77)

112: "SLR-adjusted" I assume this means removing the linear trend, but it's not defined explicitly and could be confusing.

The sentence is meant to convey that the NTR distribution is combined with the tide predictions and SLR trend to generate a predicted water level distribution. The sentence has been clarified and now reads:

> “The predicted NTR distribution is then combined with the tide predictions and SLR trend and compared to an input flood threshold…” (L104-105)

132-134: I think this paper is using the p(t) term, but this sentence confused me. Can you be explicit that it is included?

We have added an explicit clarification that this paper is indeed using the p(t) term (the persistence model). (L125)

146-153: worth including some details about the offshore boundary conditions, forcing, and source of linear trends here.

As suggested, we have added details about offshore boundary conditions (L141-143), model forcing (L143-146), and the source of linear trends (L146-150).

208-209: Are these sometimes dry locations used in the analysis, and which are they?

We have now specified explicitly the names of the stations which were extremely rarely (<0.1 %) dry. We have also modified the sentence to make it clearer that these three stations are retained in the analysis. The sentence now reads:

> “We considered 61 stations in total. The CORA timeseries always indicated “wet” conditions (no missing data) at 58 of these 61 considered stations; at the remaining

> three stations- Apalachicola, F.L.; Bay Waveland Yacht Club, M.S.; and Port Isabel, T.X.- CORA data were dry <0.1% of the training period.” (L207-210)

213-214: note smaller fraction of unassimilated than assimilated. And note the spatial distribution.

As suggested, we have added explicit notes that more stations are assimilated than are not and have noted the spatial distribution. The sentence now reads:

> “Of the 61 considered stations, more were assimilated in CORA (38) than were not (23). Assimilated stations are in open-coast environments, while non-assimilated stations are not.” (L215-216)

278-279: this sentence confused me. perhaps could be dropped?

As suggested, we have dropped this confusing sentence. We have added the relevant details to the next sentence, which now reads

> “To adjust the (trendless) tide predictions for the observed SLR trend, [25] calculated a linear trend from 1980 through 2019 at each NWLON site following [36].” (L277-278)

293: "generalize these likelihood categories" -- could you remove?

As suggested, we have removed this wording (L288-292).

371-382: units for bias and MAE

Hazard level MAE and bias do not have typical units: for example, MAE of 0.1 indicates a mean absolute error of 10% of a hazard level. To clarify the units, in the initial description of the metrics in the Methods, we have added the following example:

> “For example, a HL MAE of 0.1 indicates an average absolute HL error of 10% of a hazard level.” (L297-298)

465-493: I think the inclusion of time series at two representative locations, for each application, would help the reader understand what was happening to modify the HLs.

We thank the Reviewer for this helpful idea. Rather than creating a new figure to show example timeseries with HLs, we decided to include HL information in the example timeseries already shown in what is now Figure 3. We feel these five examples show a nice range of HL statistics and help to build an intuitive understanding of the HLs, as the Reviewer suggests.

493: is "morphodynamics" the right word? hydro- instead?

(Note that this comment appears to be concerning original line 475)

Yes, we feel that “morphodynamics” is the correct word to use here. Ocean-driven flooding results from the interactions and feedbacks between hydrodynamics and morphology, which is the core definition of morphodynamics. We feel it is important to capture both the hydrodynamic

component and the morphological component (e.g. consolidated sediment in an estuary vs. an open sandy coast with beach berms and dunes) when discussing drivers of flooding. We have retained the word “morphodynamics”.

511 and 526: can you use a better word than "strong"?

(Note that this comment appears to be concerning original lines 503 and 518)

We have replaced these two uses of the subjective “strong” with specific, quantitative language summarizing the results. (L513-514 and L530-532).

511-534: here is where substantive issues 1 and 2 might be discussed in more detail, if not earlier in the results section.

As described in detail in our responses to substantive issues 1 and 2, we have added discussion on these points.

590-593: see substantive issue #4.

Please see our response to substantive issue #4.
